# A Pilot Study: The Efficacy of Virgin Coconut Oil as Ocular Rewetting Agent on Rabbit Eyes

**DOI:** 10.1155/2015/135987

**Published:** 2015-02-23

**Authors:** Haliza Abdul Mutalib, Sharanjeet Kaur, Ahmad Rohi Ghazali, Ng Chinn Hooi, Nor Hasanah Safie

**Affiliations:** Programme of Optometry & Vision Sciences, School of Healthcare Sciences, Faculty of Health Sciences, Universiti Kebangsaan Malaysia, Jalan Raja Muda Abdul Aziz, 50300 Kuala Lumpur, Malaysia

## Abstract

*Purpose*. An open-label pilot study of virgin coconut oil (VCO) was conducted to determine the safety of the agent as ocular rewetting eye drops on rabbits. *Methods*. Efficacy of the VCO was assessed by measuring NIBUT, anterior eye assessment, corneal staining, pH, and Schirmer value before instillation and at 30 min, 60 min, and two weeks after instillation. Friedman test was used to analyse any changes in all the measurable variables over the period of time. *Results*. Only conjunctival redness with instillation of saline agent showed significant difference over the period of time (*P* < 0.05). However, further statistical analysis had shown no significant difference at 30 min, 60 min, and two weeks compared to initial measurement (*P* > 0.05). There were no changes in the NIBUT, limbal redness, palpebral conjunctiva redness, corneal staining, pH, and Schirmer value over the period of time for each agent (*P* > 0.05). *Conclusion*. VCO acts as safe rewetting eye drops as it has shown no significant difference in the measurable parameter compared to commercial brand eye drops and saline. These study data suggest that VCO is safe to be used as ocular rewetting agent on human being.

## 1. Introduction

Dry eye is multifactorial disease of the tears and ocular surface that results in symptoms of discomfort, visual disturbance, and tear film instability with potential damage to the ocular surface. It is accompanied by an increased osmolality of the tear film and inflammation of the ocular surface [1].

Tear volume is essential for many functions in maintaining the overall ocular health hence giving clarity vision [2]. Dryness of the eye will have a major impact on the ocular health and visual clarity especially in contact lens wearers [3].

Among all the therapeutic options for dry eye disease, artificial tears are still the mainstay in the initial management of a dry eye patient. Due to the complex nature of the tear film, it is difficult to design an artificial tear solution that is identical to human tears. However, many artificial tear brands have been tried to improve their quality by altering the composition, viscosity, and/or osmolarity of the solution. Though many of these rewetting drops are available over the counter, many were known to cause only temporary relief from signs and symptoms or may not even be an effective 3 treatment [4] and some of these rewetting agents have not been tested for its efficacy scientifically.

Several brands of artificial tears commercially available contain hydroxypropyl methylcellulose (HMC), hydroxypropyl guar (HP guar), sodium hyaluronate (SH), polyvinyl alcohol (PVA), and oil-based tears. These compositions of artificial tears have been tested and well discussed of its potential use in specific causes of dry eye disease [5].

A study in the literature showed the liposomal spray which was applied to the closed eye had increased the thickness of the lipid layer and improved tear film stability significantly. This brought an idea of usage of oil able to act as supplement for the tear film.

Virgin coconut oil (VCO) is obtained from the fresh, mature kernel of the coconut in which the oil extraction does not involve the use of thermal or chemical treatments [6]. VCO is emerging functional food oils due to its ability to possess several biological activities such as antiviral and antimicrobial [6]. Current findings revealed that virgin coconut oil (VCO) has been used extensively as supplement in many medical treatments. These benefits of coconut oil can be attributed to the presence of lauric acid, capric acid, and caprylic acid, and its properties such as antimicrobial, antioxidant, antifungal, antibacterial, and soothing. In addition, virgin coconut oil is also composed mainly of medium-chain triglycerides, which may not carry the same risks as other saturated fats [7].

A new horizon in discovery of the benefits of VCO has been emphasized in most systemic diseases either in curing or in treating at the same time. But none has been explored on its effects on ocular health. The possibility of using VCO as one of the remedies for dry eyes should be looked into for its efficacy and effects on short and long term basis.

In this study, three rewetting agents will be used: virgin coconut oil (VCO) which is oil-based tears, Tears Naturale II which is readily available commercially which contains HMC (hydroxymethylcelluose), and saline for control. The general aim of this pilot study is to evaluate the efficacy and safety of VCO as ocular rewetting agent on rabbit eyes.

## 2. Materials and Methods

### 2.1. Subjects

Nine female white albino New Zealand rabbits weighing between 1.5 and 2.0 kg were used for this study. The rabbits were quarantined and acclimatized for two weeks before the experiments in the Animal Laboratory Programme Biomedical Science, National University of Malaysia (UKM), Kuala Lumpur campus, purposed for adaptation period. Experiments were performed on the rabbits under standard conditions throughout the study as follows: room temperature 26°C ± 4°C, relative humidity 60% ± 10%, and alternating 12-hour light-dark cycles (8 AM to 8 PM). They were fed with standard diet and tap water. Exclusion criteria were presence of any ocular disease, In this study all care and handling of rabbits was done with approval of the institutional authority for laboratory animal care and approval from the animal ethics committee, number for ethics: FSK/OPTO/2012/HALIZA/12 DEC./482-DEC.-2012-MAY-2013.

Nine rabbits were randomly assigned to one of the three different eye drops on the tested eye. The contralateral eye was used as control. The eye drops that were used in this study were VCO, Tears Naturale II (commercial brand), and saline. Each eye drop was instilled into a small bottle which has been labelled as agents I, II, and III. The content of each bottle was masked to researcher who was responsible for instillation of the eye drops into rabbit's eye. In both tested eye and controlled eye, same parameters such as NIBUT, anterior eye assessment, corneal staining, pH, and tears production were measured before instillation as baseline reading at 0 minutes 15 and 30 and 60minutes after instillation.

In all animals, drops were instilled topically in tested eye, three times daily starting at 8 AM for the duration prescribed for each rabbit which is 14 days. Postmeasurement of that parameter was done after 14 days.

### 2.2. Parameters Measured

#### 2.2.1. NIBUT (Noninvasive Break-Up Time)

NIBUT was measured with a hand-held tearscope-plus. The lids were blinked manually to distribute the tear film and then the eye was held open and the time taken for distortion of the reflected image of the tearscope grid was recorded. In each measure, the test was performed three times successively and the average was calculated.

#### 2.2.2. Anterior Assessment

Anterior eye of the rabbit was examined by using burton lamp with cobalt blue filter. The anterior eye which included limbal redness, conjunctiva redness, and palpebral conjunctiva was assessed based on the Efron Grading Scale. The score of this test was graded from 0 to 4 (0 = normal; 1 = trace; 2 = mild; 3 = moderate; 4 = severe).

#### 2.2.3. pH

pH strip (Macherey-Nagel) was inserted into fornix (between the upper palpebral conjunctiva and cornea). The pH value was assessed by using two colors coding indicator on the strip. The particular pH value then can be determined from the respective colour reaction which is a reaction of substance that causes a colour change on the color coding. pH value was recorded in two decimal values.

#### 2.2.4. Cornea Staining

Fluorescein strip which was dipped into a drop of saline solution sodium was instilled in the conjunctival sac. The eye was examined using a burton lamp with cobalt blue light. The pattern of cornea staining was assessed based on the Efron Grading Scale. The score of this test was graded from 0 to 4 (0 = normal; 1 = trace; 2 = mild; 3 = moderate; 4 = severe).

#### 2.2.5. Schirmer's Test

STT-1 was performed without topical anesthesia. The Schirmer tear testing paper (Clement Clarke International Limited) was used for the STT-1. One sterile Schirmer tear test paper was inserted carefully into the lower conjunctival fornix of each eye at the anterior-media one-third of the eyelid. The test paper was left with the eye closed manually for 60 seconds and then removed. The amount of wetness on the test paper was immediately measured against a standard scale calibrated in millimeters and recorded in two decimal values.

### 2.3. Statistical Analysis

All results were expressed as mean ± SD. The experimental analyses of the data were carried out by one-way analyses of variance (ANOVA). Statements of statistical significance are based on *P* < 0.05. These analyses were accomplished by using statistical analyses system configured for computer (SPSS 20.0).

## 3. Results and Discussion

The mean ± SD of the noninvasive break-up time (NIBUT); anterior segment assessment, limbal redness, conjunctival hyperemia, and palpebral conjunctival hyperemia; pH value; corneal staining; and Schirmer value was shown in Tables 1 to 2. The data was analysed by the nonparametric Mann-Whitney *U* test for intragroup variations of the control and tested eyes. In addition, nonparametric Friedman test was used to analyse the changes of different parameters in certain period of time of each agent.

The baseline readings of the NIBUT in the tested eye and control eye group are shown in Tables 1 and 2, respectively. The baseline NIBUT measurements were mean ± SD: 20.73 ± 1.69 sec, 21.99 ± 2.13 sec for the control (Table 2) and study eye (Table 1) respectively. These baseline readings of NIBUT for both eyes were not significantly different (*P* > 0.05). No significant difference was found in baseline measurements of corneal staining, limbal redness, conjunctival hyperemia, and palpebral conjunctiva between the control eye and tested eye (*P* > 0.05).

**Figure 1:**
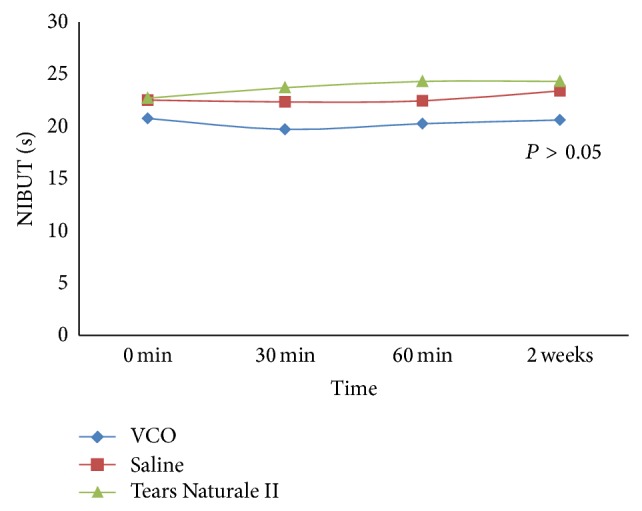
Changes of NIBUT value for tested eye.

After instillation of a drop of VCO, Tears Naturale II, and saline, there was no significant increase in tear film stability at 30 min, 60 min, and 2 weeks in comparison with baseline distribution (*P* > 0.05, Figure 1). Furthermore, after treatment of VCO, Tears Naturale II, and saline, there was also no significance difference found in the corneal staining, limbal hyperemia, and palpebral hyperemia at 30 min, 60 min, and 2 weeks in comparison with baseline distribution (*P* > 0.05). Even though VCO and Tears Naturale II did not show significance difference in the conjunctival hyperemia (*P* > 0.05) over the period of study, saline treated eyes showed a significant increase (*P* < 0.05) in the conjunctival hyperemia over the period of study. However, further statistical analysis showed the saline studies eye had no significant difference (*P* > 0.05) over the baseline readings. In treatment with VCO, it was noticed that it was slightly irritant to rabbit eyes, causing transient inflammation on the eyelid. The rabbits were annoyed and some itching of eyelids was observed for 1 minute after application of VCO.

In this study, Schirmer test also showed no significant difference between Schirmer mean values during pre and postmeasurement. The Schirmer mean value after VCO is instilled in tested eyes was of 0.67 ± 0.58 mm (after 30 minutes), 0.83 ± 0.76 mm (after 60 minutes), and 1.00 ± 0.50 mm (after 2 weeks), respectively, *P* > 0.05, as shown in Table 1.

Results of the pH measurements for all the agents studied on both eyes are as follows. Saline and Tears Naturale II had pH values ranging from 8.4 to 8.6; meanwhile VCO was constant with pH value of 8.6 over the test period. Table 1 describes the pH of all agents on each time point on left eyes. There were no significant differences in pH values between all three agents along this study conducted, *P* > 0.05. Although Tears Naturale II and saline showed some differences in pH values after the instillation, the pH change were not significant (*P* > 0.05) when the Friedman statistical analysis done. A previous study had indicated that VCO was an efficient way in treating eyelid puffiness.

The main aim of this study was to determine the efficacy and safety of VCO as a rewetting agent on the anterior segment eyes of rabbits. There was a review which indicated that VCO was an efficient way for treatment of puffy eyelids. However, there was no research which shows its efficiency as a rewetting eye drop. In this study, NIBUT, corneal staining, anterior segment assessment, Schirmer-1 test, and pH test were used to determine the efficacy of the VCO. This was quite similar to another study which measured the efficiency of tear substitutes by relief of symptoms, decrease in tear film break-up time, decrease in fluorescein staining intensity, and improvement in Schirmer wetting [7]. The measurement of NIBUT value is able to provide index of efficiency which is more effective [8]. In addition, measurement of NIBUT value provides sensitivity of 82% and specificity of 86% in classification of dry eyes [9].

The topical applications of VCO were found to have no significant difference in NIBUT in comparison to the control eye for both 30 and 60 minutes and even after two weeks' time. At the same time, VCO was found with no statistical difference in NIBUT with the other tested agents. This indicates that the application of VCO did not cause any disturbance in the tear film layer. In this case, the mean ± SD NIBUT value of 20.73 ± 1.69 sec found in this study was different with the research finding which indicates the mean ± SD NIBUT value for normal rabbit 29.80 ± 3.40 min [10]. However, there was a research which compared the two cyclooxygenase inhibitors in an experimental dry eye model in albino rabbits which revealed that the baseline NIBUT for the control group was 18.80 ± 5.20 sec [11]. This is similar to the outcome findings for the baseline NIBUT of the control group in our study. Even though there is a conflict in the average NIBUT value for a rabbit with good ocular health, we can show that the VCO does not cause any harmful effect to the rabbit's eye.

According to Bron et al. [12] fluorescein staining is an effective method for ocular surface evaluation. Fluorescein staining results from the disruption of corneal epithelial cell to cell junctions or damaged corneal epithelial cells [13]. In this research, corneal staining did not show any significant difference after the instillation of all types of eye drop (*P* > 0.05). However, we found that all the rabbits showed some mild staining even in the baseline measurement. Some staining was observed on the surface of the cornea in nearly all the cases of normal rabbits [14]. In addition, there is a correlation in corneal staining phenomenon in both eyes of the rabbit. It was concluded that the fluorescein staining phenomenon was related to physiological desquamation of the corneal epithelium which was normal.

In this research, anterior eye segment was divided into three parts, namely, limbal redness, conjunctiva redness, and palpebral redness. Instillation of VCO and Tears Naturale II did not play any significant difference in the above anterior eye segments. However, it revealed significant changes in conjunctiva redness only after 30 min and 60 min instillation of saline. This phenomenon can be explained as it was probably caused by the process of pH measurement. During pH measurement, it had been observed that the rabbit's eye turns red once the pH strip was inserted into the upper bulbar conjunctiva; however, the conjunctival redness reduced during the postmeasurement.

A cohort study had shown there were no correlations between dry eye symptoms and conjunctival and corneal staining, as well as bulbar and limbal hyperaemia in dry eye patient [12]. These findings were similar with current literature which indicated poor relationships between ocular signs and dry eye symptoms. Further investigation is needed to find out the main causes. VCO which acts as a rewetting agent will not influence the anterior segment of the eyes other than the changes of the conjunctiva redness.

Schirmer values for all rabbits were in normal range 0.67–1.00 during baseline (0 minutes). According to previous report, normal mean values for Schirmer-1 on New Zealand rabbits breed were 8.82 ± 3.52 mm [15]. These showed that all the three subjects eyes were in healthy condition (they did not have dry eyes). Based on the data, VCO, as well as Tears Naturale II and saline, did not affect the quantity of tears. This result could be explained by the content of fatty acids in VCO called medium-chain triglyceride (MCT). MCTs act in lipid layer as internal phase, which link the drug in ophthalmic emulsion to stabilize tear layers and eye surface to treat dry eye more effectively than topical eye drops based on aqueous products [16]. However, tear film on rabbit eye was believed to be more stable than human's tears due to higher concentration of divalent cations (e.g., Magnesium and Calcium ions) found in aqueous layer. These positive ions will interact with negatively charged ions in lipid layer which theoretically forms cross-linking to stabilize the tear layers [10]. Their study found that divalent cations have strong influence on rabbit tears but not in human tears.

The tear pH in this study was found to be more alkaline (between 8.4 and 8.6) as compared to human tears before instillation of agents (at 0 minutes). The pH value of human tears in previous study was 7.1 ± 1.5 and it was assumed to be influenced by time [17]. In the study tear pH will shift to being alkaline during the day (due to eyes opened) and will shift to being acidic after eyes were closed for an hour. This phenomenon could be explained by bicarbonate buffer system in tears and tear layers which contained lipid, mucin, and aqueous layer. The condition of rabbit eye (widely opened all the time) and measurements taken during daytime may cause the result obtained in this study to be alkaline in rabbit tears.

In this study, VCO had shown no influence in pH values in different periods of time. Study about tear pH done by Coles and Jaros [17] indicated that although the acidic solution (Mydriacyl, pH 4.8, and Phenylephrine, pH 6.0) is instilled into the eyes, the acidic tears will return to initial pH for about 20–40 minutes due to tear reflex. The previous study of pH in solution (for cleaning contact lenses) showed none of them correspond to tear pH. However, no physiological changes should occur in the normal eye [18]. There were differences in the pH of tears due to factors of carbon dioxide and meibomian lipids.

All animals appeared normal on examination by naked eye and with a burton lamp. However, it was noticed that agent VCO was slightly irritant to rabbit eyes, causing transient inflammation on the eyelid. The rabbits were annoyed and some itching was observed for 1 minute after application of agent VCO. The exact cause for this phenomenon was unknown. However, it may be due to the overflow of the oil-based drop causing the fur on surrounding eyelid to clump together and resulting in the uncomfortable feeling. Higher viscosity in agent could be the reason which causes this effect. High viscosity eye drops may induce unwanted visual disturbance [19] and precipitate as crystals on eyelids and lashes. Since the VCO was used on rabbits with normal eye physiology, the rabbits did not show any sign of hyperemia or significant changes in the parameters measured. It is expected that the oil will likely seep into the cornea which could affect vision if the subjects have dry eyes and furthermore would disturbs the physiology of the eye. The dripping is not a major concern if it is being used in human as previous study [20] has shown that it is safe on the human skin. However further study is needed to be carried out to investigate the suitable volume of VCO to be used as ocular rewetting agent in rabbits.

Instillation of vital dyes such as fluorescein is one of the invasive methods to diagnose dry eyes. It is important to perform them systematically so that the interaction and influence to the outcome of the following test can be minimized [20]. In this research the usage of fluorescein has been done after the assessment of NIBUT, pH, and the anterior segment of eyes.

## 4. Limitation

There were some unavoidable problems found in this study. Firstly, due to financial constraints, the population size used in this research was small (*N* = 9). Statistically, research was analysed using nonparametric statistics with a defined level of confidence. The sample size must be adequate to demonstrate superiority. In this study, the small samples size used could disturb the final analysis.

In this research, it was found that the restrainer used is too big for the rabbits where the rabbits still have rooms to hide. In the study, the rabbit's eye was examined in a condition without restrainer to reduce stress symptoms for rabbits. Due to this reason, the time taken for researchers to examine the rabbit's eye was longer as the researchers need to calm the rabbit down each time. Hence the measurement was collected at an extended time.

The fluorescein used to assess the corneal staining pattern is considered as an invasive method. Fluorescein tends to affect the pH value of the tears. In this research, pH of the tears is measured before the corneal staining pattern assessment. However, it might cause some carry-over effect on the pH measurement in 30 and 60 minutes after the instillation of drop. Irrigation of fluorescein is not possible as it might interfere with the effect of eye drops. Hence, this problem is unavoidable because the pH parameter needs to be measured after 30 and 60 minutes.

Based on the limitations, there are a few suggestions for future research. This can be done by increasing the sample size to enhance the strength of the statistical analysis. Besides that, duration of the study should be extended to ensure the efficacy. A favourable method to collect data should be improvised in order to speed up the procedure. Lastly, the procedure during data collection should be planned well so that the limitations can be avoided.

## 5. Conclusions

In comparison with Tears Naturale II and saline, VCO which acts as a rewetting agent did not cause any significant difference in NIBUT, corneal staining pattern, anterior segment, Schirmer Test and pH in 30 min, 60 min, and 2 weeks after instillation. VCO is not merely a composition of fatty acids but it also acts as a protective layer over the tear film layers from evaporation. VCO with its anti-inflammatory properties might be useful for those with dry eyes problem. However, further investigation should be done to determine its efficacy for dry eyes therapy. In conclusion, it has been shown that VCO did not cause harmful effects when used on rabbits' eyes. This finding suggests that VCO are safe to be used on human's eyes. Thus, future research on human needs to be conducted to study the efficacy of VCO as rewetting agent on dry eye patient. The beneficial effect of VCO is most likely attributed to its anti-inflammatory properties, which is similar to those of natural tears.

## Figures and Tables

**Table 1:** Mean (±SD) for study eyes at each time point using different types of drops. (Agent I: VCO, agent II: saline, and agent III: Tears Naturale II.)

Mean (±SD)	Agent	0 min	30 min	60 min	2 weeks
NIBUT	I	20.76 ± 2.36	19.71 ± 2.10	20.26 ± 2.26	20.61 ± 1.96
	II	22.52 ± 2.60	22.34 ± 3.59	22.44 ± 3.05	23.40 ± 3.30
	III	22.70 ± 1.57	23.71 ± 2.24	24.31 ± 3.03	24.31 ± 3.29

Corneal staining	I	0.83 ± 0.29	1.00 ± 0.00	1.00 ± 0.00	1.00 ± 0.00
	II	1.00 ± 0.00	1.00 ± 0.00	1.00 ± 0.00	1.00 ± 0.00
	III	1.00 ± 0.00	1.00 ± 0.00	1.00 ± 0.00	0.83 ± 0.29

Limbal hyperemia	I	0.83 ± 0.29	1.00 ± 0.00	1.00 ± 0.00	1.00 ± 0.00
	II	1.00 ± 0.00	1.00 ± 0.00	1.00 ± 0.00	1.00 ± 0.00
	III	1.00 ± 0.00	1.00 ± 0.00	1.00 ± 0.00	0.83 ± 0.29

Conjunctival hyperemia	I	0.83 ± 0.29	1.33 ± 0.29	1.50 ± 0.00	1.00 ± 0.00
	II	0.67 ± 0.58	1.50 ± 0.00	1.50 ± 0.00	0.50 ± 0.50
	III	1.00 ± 0.00	1.00 ± 0.00	1.00 ± 0.00	0.83 ± 0.29

Palpebral hyperemia	I	0.83 ± 0.29	1.33 ± 0.29	1.50 ± 0.00	1.00 ± 0.00
	II	0.67 ± 0.58	1.50 ± 0.00	1.50 ± 0.00	0.50 ± 0.50
	III	1.00 ± 0.00	1.17 ± 0.29	1.33 ± 0.29	0.83 ± 0.29

Schirmer test	I	1.00 ± 0.00	1.33 ± 0.29	1.33 ± 0.29	1.00 ± 0.00
	II	0.67 ± 0.58	0.67 ± 0.58	0.83 ± 0.76	1.00 ± 0.50
	III	1.00 ± 0.00	1.00 ± 0.00	1.00 ± 0.00	0.83 ± 0.29

pH	I	7.67 ± 0.19	7.67 ± 0.38	7.89 ± 0.22	8.00 ± 0.39
	II	7.34 ± 1.45	7.67 ± 0.38	7.67 ± 0.19	7.56 ± 1.06
	III	6.66 ± 0.88	6.67 ± 0.96	7.22 ± 0.73	7.11 ± 0.22

**Table 2:** Mean (±SD) for control eyes at each time point without drops.

Mean (±SD)	0 min	30 min	60 min	2 weeks
NIBUT	19.65 ± 1.53	19.60 ± 1.49	20.63 ± 2.06	19.81 ± 1.89
	20.82 ± 1.37	20.63 ± 1.75	20.71 ± 2.43	19.28 ± 1.20
	21.74 ± 1.99	20.33 ± 1.41	19.85 ± 1.64	20.80 ± 0.92

Corneal staining	0.83 ± 0.29	0.83 ± 0.29	0.83 ± 0.29	0.83 ± 0.29
	1.00 ± 0.00	1.00 ± 0.00	1.00 ± 0.00	0.83 ± 0.29
	1.33 ± 0.58	1.33 ± 0.58	1.33 ± 0.58	0.83 ± 0.29

Limbal hyperemia	0.83 ± 0.29	0.83 ± 0.29	0.83 ± 0.29	0.83 ± 0.29
	1.00 ± 0.00	1.00 ± 0.00	1.00 ± 0.00	0.83 ± 0.29
	1.33 ± 0.58	1.33 ± 0.58	1.33 ± 0.58	0.83 ± 0.29

Conjunctival hyperemia	0.83 ± 0.29	1.00 ± 0.00	1.17 ± 0.29	1.00 ± 0.00
	1.00 ± 0.00	1.33 ± 0.29	1.33 ± 0.29	0.50 ± 0.50
	1.00 ± 0.00	1.17 ± 0.29	1.17 ± 0.29	1.00 ± 0.00

Palpebral hyperemia	0.83 ± 0.29	1.00 ± 0.00	1.17 ± 0.29	1.00 ± 0.00
	1.00 ± 0.00	1.33 ± 0.29	1.33 ± 0.29	0.50 ± 0.50
	1.00 ± 0.00	1.17 ± 0.29	1.17 ± 0.29	1.00 ± 0.00

Schirmer test	1.00 ± 0.00	1.00 ± 0.00	1.00 ± 0.00	1.00 ± 0.00
	1.00 ± 0.00	1.00 ± 0.00	1.00 ± 0.00	0.83 ± 0.29
	1.00 ± 0.00	1.00 ± 0.00	1.00 ± 0.00	1.00 ± 0.00

pH	7.67 ± 0.00	7.56 ± 0.59	7.56 ± 0.11	7.33 ± 0.33
	8.66 ± 0.67	8.44 ± 0.73	8.89 ± 0.29	8.00 ± 0.33
	7.11 ± 0.29	7.11 ± 0.78	7.33 ± 0.51	7.55 ± 0.22
